# Protective effect of oxytocin on LPS-induced acute lung injury in mice

**DOI:** 10.1038/s41598-019-39349-1

**Published:** 2019-02-26

**Authors:** Xiaona An, Xiaotong Sun, Yonghao Hou, Xiaomei Yang, Hongli Chen, Peng Zhang, Jianbo Wu

**Affiliations:** Department of Anesthesiology, Qilu Hospital of Shandong University, Jinan, Shandong 250012 P.R. China

## Abstract

Oxytocin (OT), a neurohypophyseal hormone synthesized in the paraventricular and supraoptic nuclei of the hypothalamus, has been reported to have an anti- inflammatory effect. However, its role in acute lung injury (ALI) has never been investigated. The aim of this study was to explore the therapeutic effects and potential mechanism action of OT on lipopolysaccharide (LPS)-induced ALI. Mice were treated with OT 30 min before the intraperitoneal injection of LPS. After 2 h, the effects of OT on lung histopathological changes, lung wet/dry (W/D) ratio, myeloperoxidase (MPO) activity, levels of inflammatory cytokines in the bronchoalveolar lavage fluid (BALF), and expression of inflammation proteins were detected. The results showed that OT significantly reduced LPS-induced pathological injury, W/D ratio, MPO activity, and the levels of interleukin (IL)-1β, IL-18 and IL-6. Further, OT also inhibited LPS-induced Toll-like receptor 4 expression and NLR family pyrin domain containing 3 inflammasome activation. OT receptor antagonist (L-368,899) was given 90 min before injecting OT to further demonstrate the role of OT in LPS-induced ALI. The results showed OT could not alleviate the aforementioned inflammatory reactions after administering L-368,899. In conclusion, the present results indicated that OT could reduce inflammatory responses of LPS-induced ALI.

## Introduction

Acute lung injury (ALI) is a critical illness syndrome with a high mortality rate of 40–60%^1^. ALI is characterized by refractory hypoxemia and progressive dyspnea. It is most often seen as part of a systemic inflammatory process. The inflammatory process was vital in the development of ALI. The injury to the alveolar epithelium and endothelium, lung edema, and infiltration of neutrophils were the main pathological changes^2,3^. Gram-negative bacterial infections are the main cause of ALI, and lipopolysaccharide (LPS), which is the main component of the cell wall of Gram-negative bacteria, is the major stimulus for the release of inflammatory mediators. LPS can also activate the host receptor TLR4 and trigger an inflammatory response, resulting in ALI^4,5^. Therefore, an effective anti-inflammatory drug used to reduce lung injury was urgently needed.

Oxytocin (OT), a neurohypophyseal hormone synthesized in the paraventricular and supraoptic nuclei of the hypothalamus, has a wide range of effects in the body. It exerts its functions via G protein–coupled receptors^6^. Besides its well-known role in uterine contraction during parturition and the milk-ejection reflex during lactation, OT is also implicated in cardiovascular regulation, body temperature regulation, feeding, gastric distension^7^, and modulation of the release of adenohypophyseal hormones^8^. OT and OT receptors (OTRs) are found in the thymus^9^ and macrophage^10^, and the OTR gene includes response elements for interleukins and acute-phase reactants^6^, indicating the involvement of OT in modulating inflammatory and immune processes. Exogenous OT administration also reduces tissue damage in a variety of animal models of injury^11,12^. Moreover, the co-administration of an OTR antagonist blocks the protective effects of OT during cardiac ischemia^12^ or cerebral ischemia in rats^13^. In addition, OT showed anti-inflammatory properties by alleviating paw edema induced by carrageenan^14^. A recent study demonstrated that OT could inhibit LPS-induced inflammation in the microglial cells and attenuate microglial activation in LPS-treated mice^15^. This study aimed to evaluate the anti-inflammatory effects and mechanism of action of oxytocin on LPS-induced ALI in a mouse model.

## Results

### OT alleviated LPS-induced histopathological changes in lungs

**Figure 1:**
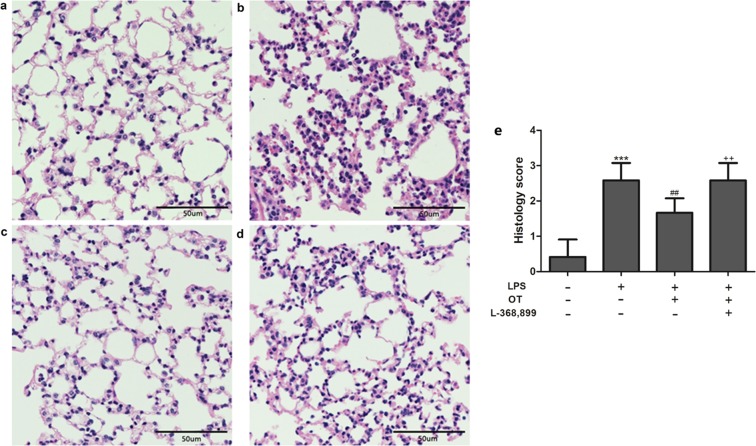
Effects of OT on the histological changes in lung tissue in mice with LPS-induced ALI. Representative histological changes in lungs obtained from mice of different groups. (**a**) Control group. (**b**) LPS group. (**c**) LPS + OT group. (**d**) LPS + OT + L-368,899 group. Pathological changes in lung tissues were observed using HE staining (light microscopy, ×200). The histopathologic scores are presented for the lung tissues (**e**). The data are presented as the means ± standard error of the mean. ***P < 0.001 vs control. ^##^*P* < 0.01 vs LPS. ^++^*P* < 0.01vs OT. Data show means with SEM for six mice/group.

The pathological changes were detected using HE staining to investigate the protective effects of OT on LPS-induced ALI. As shown in Fig. 1, lung tissues from the control group revealed a normal structure and no histopathological changes under a light microscope (Fig. 1a). LPS group exhibited; (Fig. 1b). However, LPS-induced pathological changes were significantly attenuated by OT (Fig. 1c). However, L-368,899 obviously aggravated histopathological changes in lungs compared with the OT group (Fig. 1d). And histological lung injury scores are presented (Fig. 1e).

### Effects of OT on MPO activity

**Figure 2:**
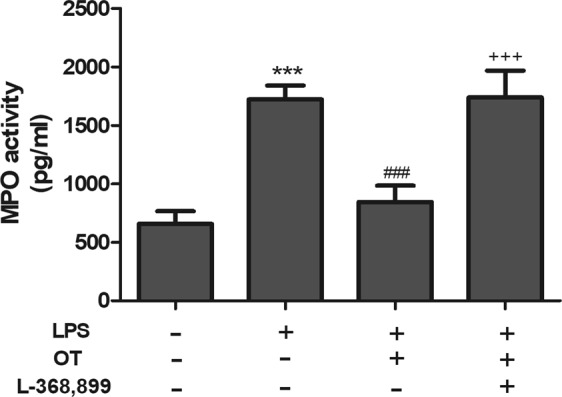
Effects of OT on lung MPO activity in mice with LPS-induced ALI. The values are presented as means ± standard error of the mean. ***P < 0.001 vs control. ^###^*P* < 0.001 vs LPS. ^+++^*P* < 0.001 vs OT. Data show means with SEM for five mice/group.

The MPO activity has been known as a biomarker to assess the infiltration of neutrophils and macrophages within pulmonary tissues. As shown in Fig. 2, LPS caused a significant increase in the MPO activity compared with the control group (*P* < 0.001). Conversely, this increase was found to be significantly inhibited in the OT group (*P* < 0.001). In addition, L-368,899 administered before LPS and OT stimulation increased MPO activity compared with the OT group (*P* < 0.001).

### Effects of OT on the W/D ratio

**Figure 3:**
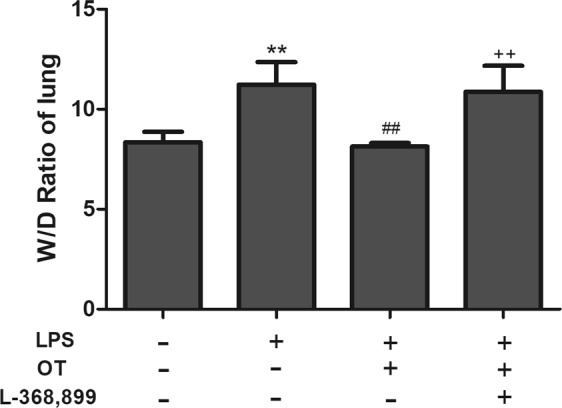
Effects of OT on the lung W/D ratio in mice with LPS-induced ALI. The values are presented as means ± standard error of the mean. **P < 0.01 vs control. ^##^*P* < 0.01 vs LPS. ^++^*P* < 0.01 vs OT. Data show means with C SEM for four mice/group.

The lung W/D ratio was evaluated to assess LPS-induced changes in pulmonary vascular permeability to water. LPS caused a significant increase in the lung W/D ratio compared with the control group (*P* < 0.01). Conversely, pretreatment with OT significantly decreased the lung W/D ratio compared with the LPS group (*P* <0.01). In addition, L-368,899 administered before LPS and OT increased the W/D ratio compared with the OT group (*P* < 0.01) (Fig. 3).

### Effects of OT on LPS-induced production of cytokines in BALF

**Figure 4:**
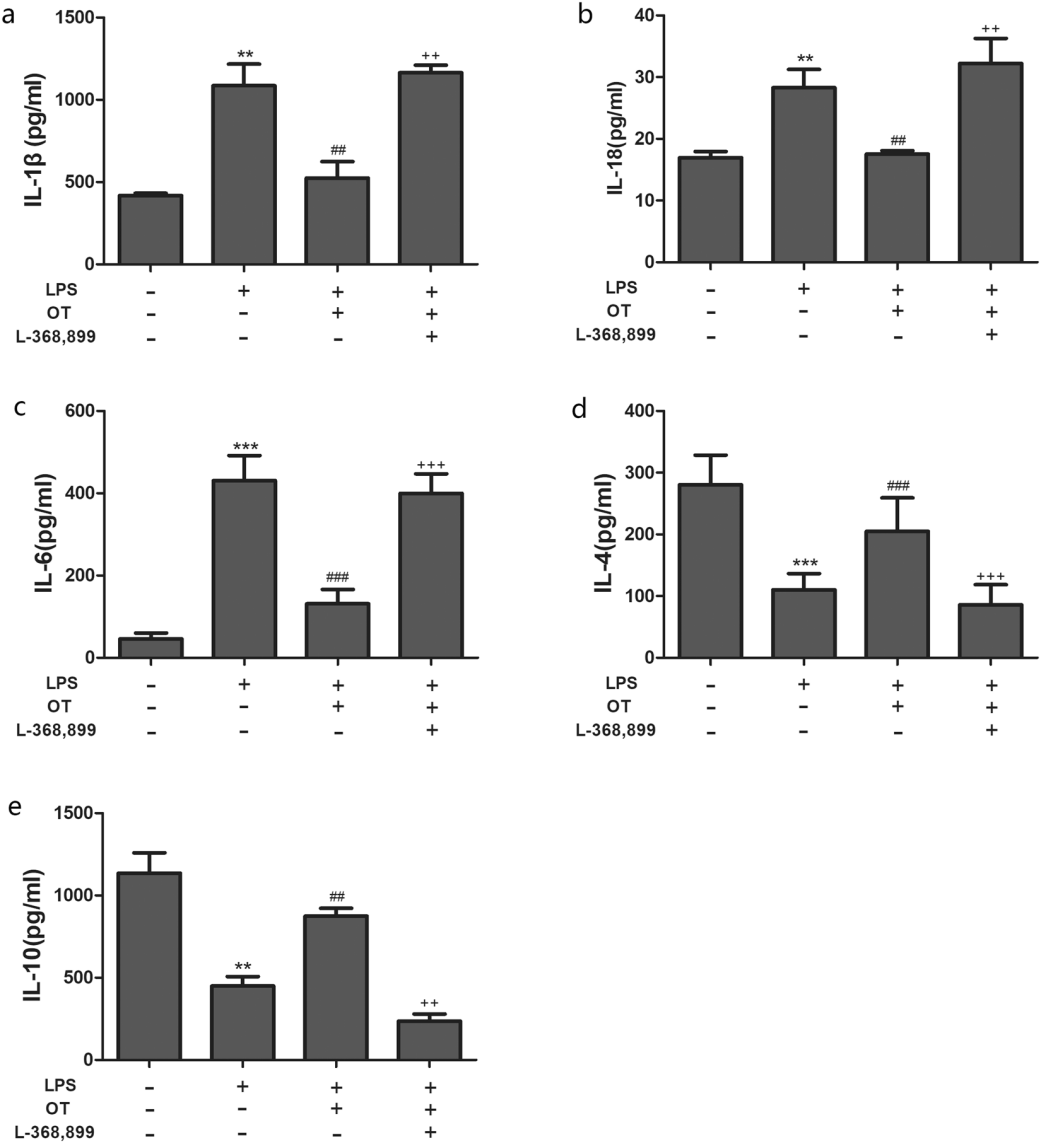
Effects of OT on the production of inflammatory cytokines in BALF from mice with LPS-induced ALI. The levels of IL-1β (**a**), IL-18 (**b**), IL-6 (**c**), IL-4 (**d**), and IL-10 (**e**) were measured using ELISA. The values are presented as means ± standard error of the mean. **P < 0.01, ***P < 0.001 vs control. ^##^*P* < 0.01, ^###^*P* < 0.001vs LPS. ^++^*P* < 0.01, ^+++^*P* < 0.001vs OT. Data show means with SEM for eight mice/group.

The production IL-1β, IL-18, IL-6, IL-4 and IL-10 in BALF was analyzed to further assess the anti-inflammatory effects of OT. As shown in Fig. 4, the levels of IL-1β, IL-18 and IL-6 were found to be significantly increased in the LPS group compared with the control group (*P* < 0.01 and *P* < 0.001). However, pretreatment with OT apparently decreased the levels of IL-1β, IL-18 and IL-6 in BALF compared with the LPS group (*P* < 0.01 and *P* < 0.001). Moreover, L-368,899 administration significantly increased the cytokines levels compared with the OT group (*P* < 0.01 and *P* < 0.001). As shown in Fig. 4, IL-4 and IL-10 levels were found to be significantly decreased in the LPS group compared with the control group (*P* < 0.01, *P* < 0.001). OT significantly increased IL-4 and IL-10 production compared with the LPS group (*P* < 0.01 and *P* < 0.001, respectively). L-368,899 administration significantly decreased IL-4 and IL-10 levels compared with the OT group (*P* < 0.01 and *P* < 0.001, respectively).

### Effects of OT on LPS-induced gene expression and protein level of cytokines in the lung tissues

**Figure 5:**
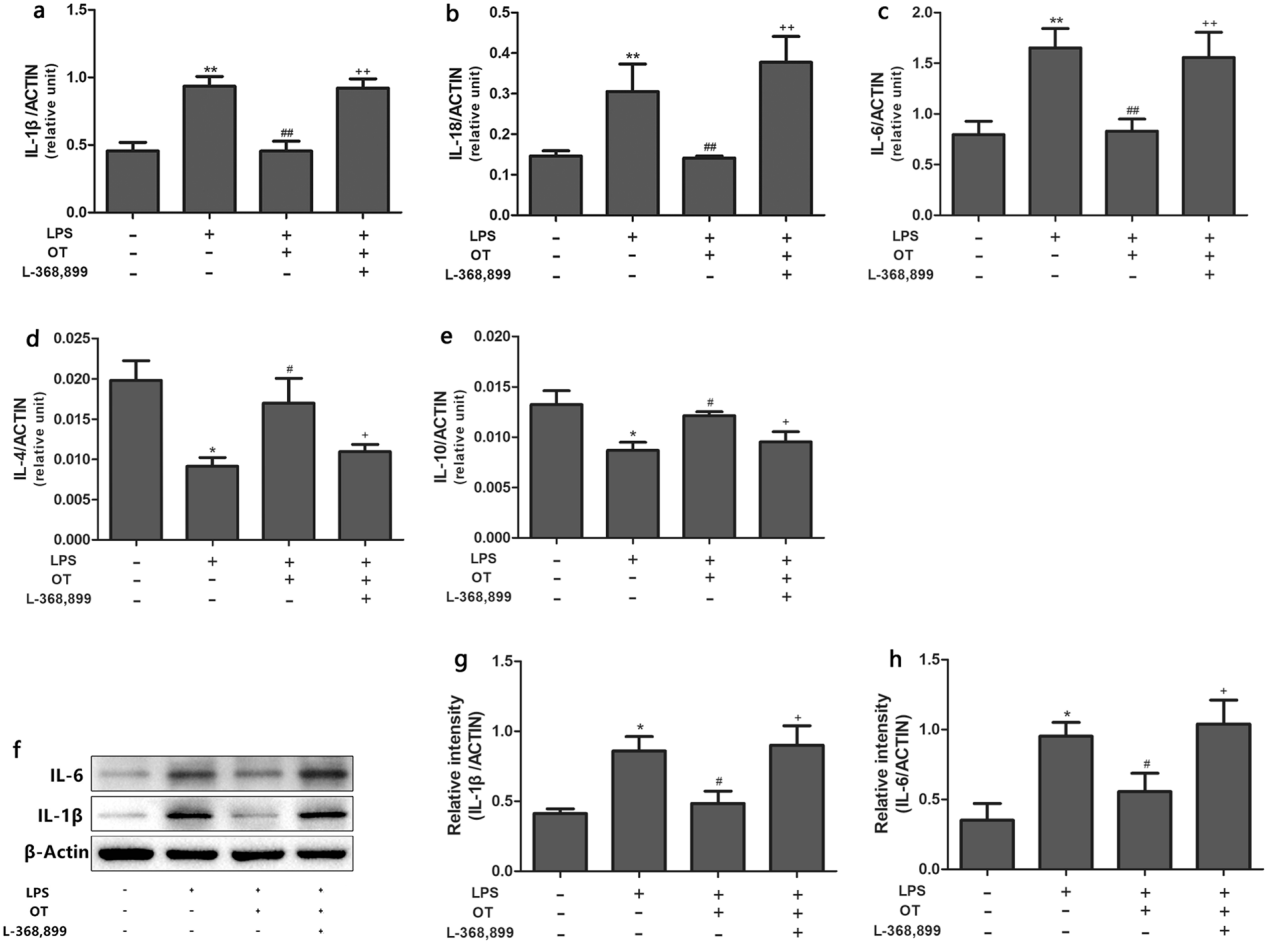
Effects of OT on the gene expression of inflammatory cytokines in the lung tissues from mice with LPS-induced ALI. The mRNA expression of IL-1β (**a**), IL-18 (**b**) IL-6 (**c**), IL-4 (**d**), and IL-10 (**e**) was measured using RT-qPCR. The proteins expression levels of IL-1β and IL-6 were analyzed using the Western blot analysis (**f**). The proteins expression of IL-1β and IL-6 were quantified by measuring band intensities and displayed as fold increase relative to ACTIN (**g** and **h**). The values are presented as means ± standard error of the mean. *P < 0.05, **P < 0.01 vs control. ^#^*P* < 0.05, ^##^*P* < 0.01 vs LPS. ^+^*P* < 0.05, ^++^*P* < 0.01 vs OT. Data show means with SEM for four to eight mice/group.

The cytokine gene expression in the lung tissues was determined using qPCR. The expression levels of IL-1β, IL-18, IL-6, IL-4, and IL-10 (Fig. 5a–e) were in agreement with the cytokines levels in the BALF (Fig. 4). Also, the proteins levels of IL-1β and IL-6 were analyzed using the Western blot analysis (Fig. 5f–h). As shown in Fig. 5f–h, the result was consistent with the cytokines levels evaluated using qPCR.

### Effect of OT on LPS-induced OTR expression

**Figure 6:**
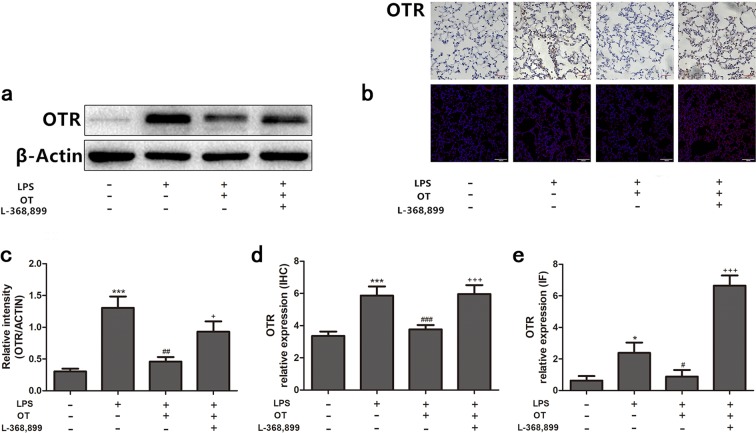
Effects of OT on the expression of OTR in the lung tissues from mice with LPS-induced ALI. The expression levels of OTR were evaluated using the Western blot analysis, IHC, and IF (**a**,**b**). The Western blot analysis, IHC, and IF of OTR were quantified respectively (**c**–**e**). *P < 0.05, ***P < 0.001 vs control. ^#^*P* < 0.05, ^##^*P* < 0.01, ^###^*P* < 0.001 vs LPS. ^+^*P* < 0.05, ^+++^*P* < 0.001 vs OT. Data show means with SEM for five mice/group.

OTR expression was detected in this study to investigate the mechanism underlying the anti-inflammatory effect of OT. As shown in Fig. 6, the protein level of OTR was increased compared with the control group. However, pretreatment with OT significantly decreased the protein expression compared with the LPS group. Moreover, L-368,899 administration significantly increased the protein expression compared with OT group. In addition, the expression of OTR was increased on macrophage in the LPS-induced ALI compared with control group (shown in Supplementary Information Fig. 2).

### Effect of OT on LPS-induced TLR4 and NF-κB expression

**Figure 7:**
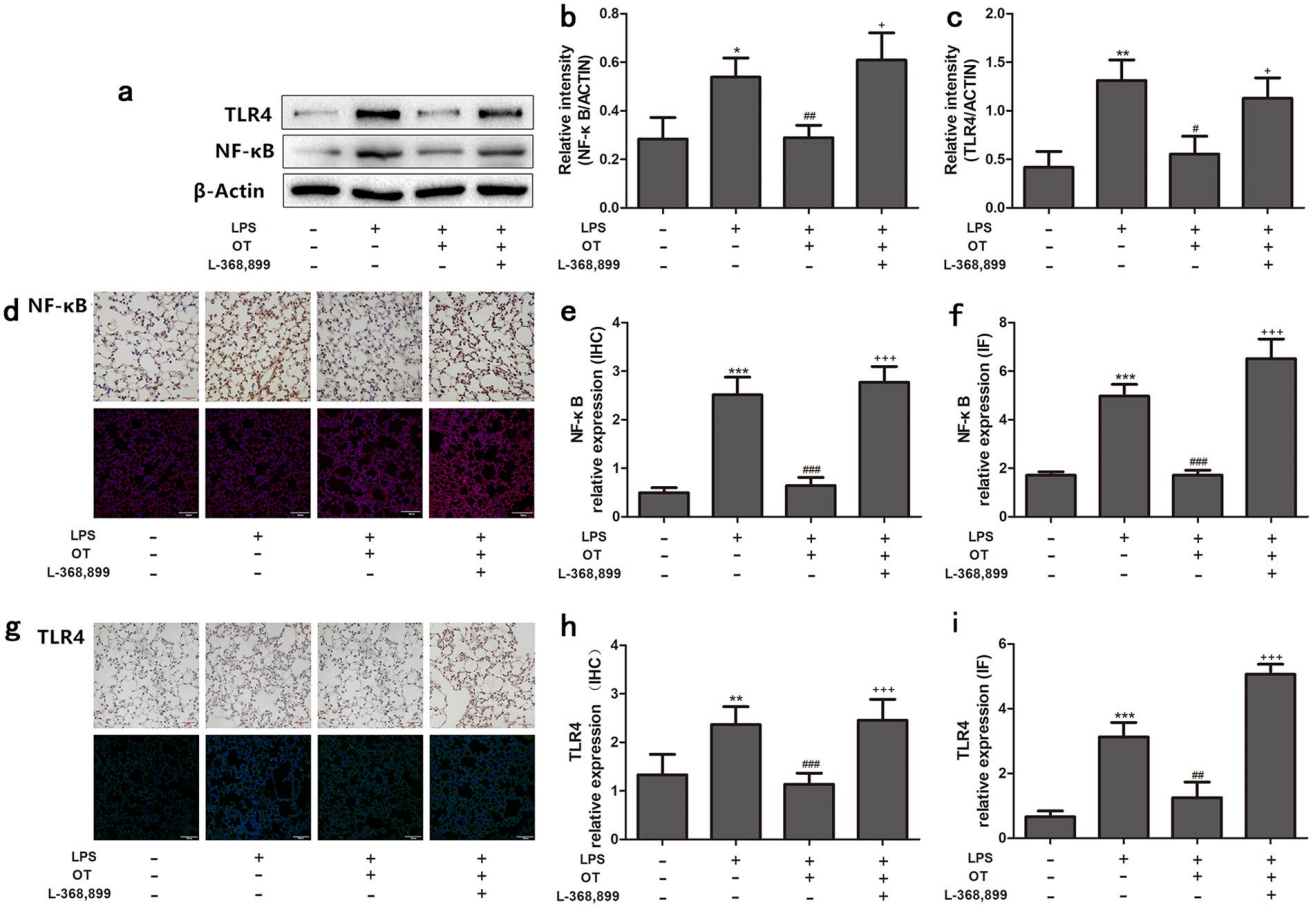
Effects of OT on the expression of NF-κB and TLR4 in the lung tissues from mice with LPS-induced ALI. The expression levels of these proteins were evaluated using the Western blot analysis, IHC, and IF (**a**,**d**,**g**). The Western blot analysis, IHC, and IF of OTR were quantified respectively (**b**,**c**,**e**,**f**,**h**,**i**). *P < 0.05, **P < 0.001,***P < 0.001 vs control. ^#^*P* < 0.05, ^##^*P* < 0.01, ^###^*P* < 0.001 vs LPS. ^+^*P* < 0.05, ^+++^*P* < 0.001 vs OT. Data show means with SEM for five mice/group.

In this study, the expression of TLR4 and NF-κB was detected using Western blot analysis, immunohistochemistry (IHC), and immunofluorescence (IF). As shown in Fig. 7, LPS significantly increased TLR4 and NF-κB expression compared with the control group. However, pretreatment with OT significantly decreased the protein expression compared with the LPS group. Moreover, L-368,899 administration significantly increased the protein expression compared with the OT group.

### Effect of OT on LPS-induced NLRP3 and caspase-1 expression

**Figure 8:**
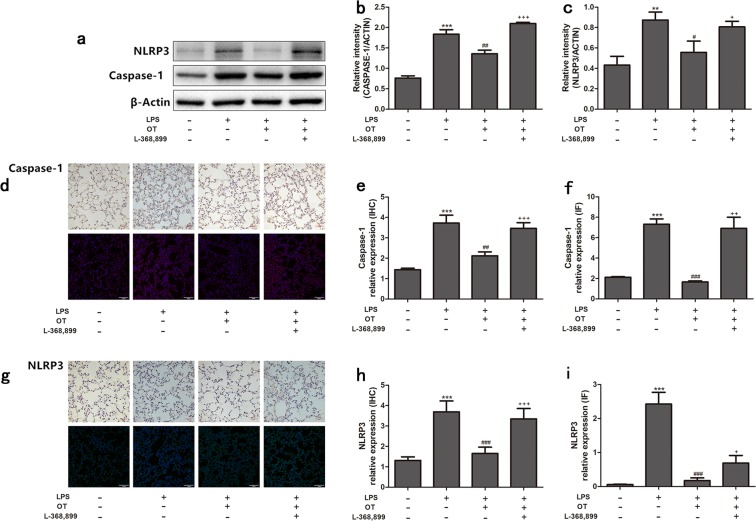
Effects of OT on the expression of NLRP3 and Caspase-1 in the lung tissues from mice with LPS-induced ALI. The expression levels of these proteins were evaluated using the Western blot analysis (A), IHC, and IF (**a**,**d**,**g**). The Western blot analysis, IHC, and IF of OTR were quantified respectively (**b**,**c**,**e**,**f**,**h**,**i**). **P < 0.01,***P < 0.001 vs control. ^#^*P* < 0.05, ^##^*P* < 0.01, ^###^*P* < 0.001 vs LPS. ^+^*P* < 0.05, ^++^*P* < 0.01, ^+++^*P* < 0.001 vs OT. Data show means with SEM for five mice/group.

The activation of NLRP3 inflammasome was detected in this study to further investigate the mechanism underlying the anti-inflammatory effect of OT. As shown in Fig. 8, LPS significantly increased NLRP3 and caspase-1 expression compared with the control group. However, pretreatment with OT significantly decreased the protein expression compared with the LPS group. Moreover, L-368,899 administration significantly increased the protein expression compared with the OT group.

### The level of OT in the lung tissue

**Figure 9:**
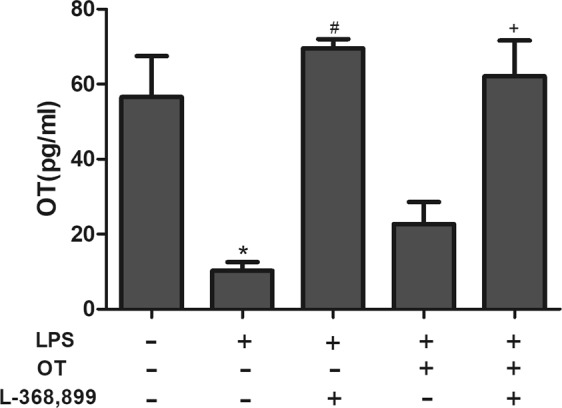
The level of OT was measured using ELISA. The values are presented as means ± standard. *P <0.05 vs control. ^#^*P* < 0.05vs LPS. ^+^*P* < 0.05 vs LPS + OT. Data show means with SEM for four mice/group.

In the study, the level of OT in the lung tissue was measured using ELISA. As shown in Fig. 9, LPS significantly deceased OT level compared with control group. However, pretreatment with L-368,899 significantly increased OT level compared with the LPS group and the LPS + OT group. These results showed that there is a feedback loop when employing the OTR antagonist in the lung. Whereas serum levels of OT are similar in all groups.

### The effect of L-368,899 on inflammatory response

**Figure 10:**
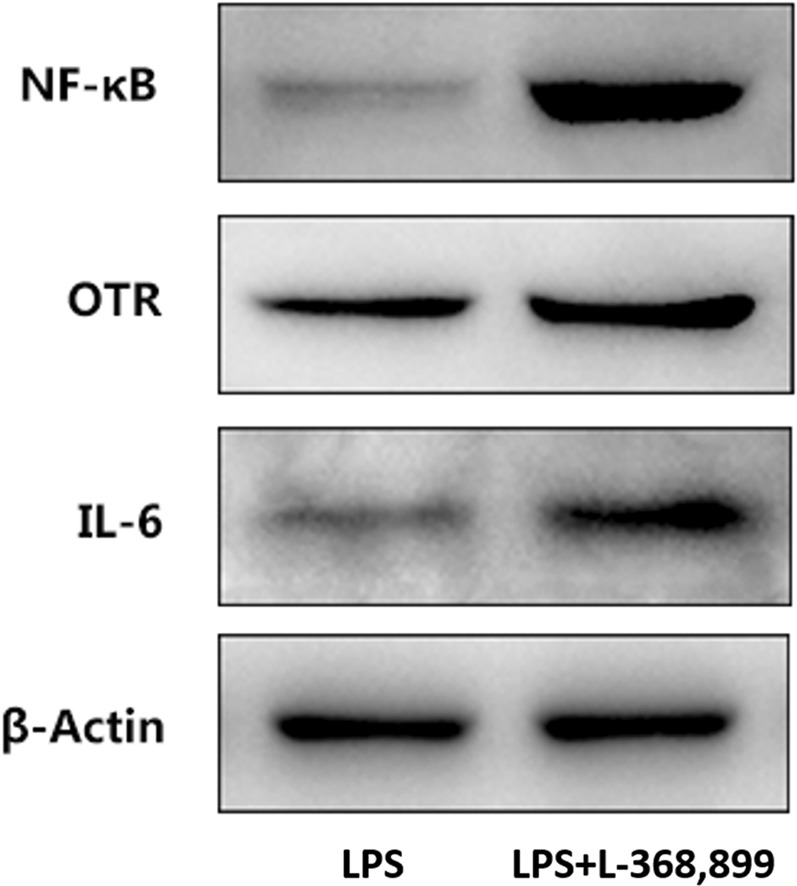
Effects of L-368,899 on the expression of IL-6, OTR and NF-κB in the lung tissues from mice with LPS-induced ALI. The expression levels of these proteins were evaluated using the Western blot analysis.

For further illustration the mechanism underlying the anti-inflammatory effect of OT, a better comparator group of LPS + L-368,899 was measured. As shown in Fig. 10, L-368,899 significantly exacerbates inflammation compared LPS group. These results suggested that oxytocin’s anti-inflammatory effects were possible due to its binding to OTR, because the anti-inflammatory effects on LPS-induced ALI were effectively blocked by the OTR antagonist L-368,899.

## Discussion

ALI has high morbidity and mortality among patients, with no effective drugs available in the clinic^16,17^. LPS-induced ALI is characterized by the release of inflammatory cytokines and expression of inflammatory proteins in the lung tissue. Previous studies demonstrated that the OTR gene contained response elements for acute-phase reactants and ILs^18,19^, and the expression of OTR was increased in LPS-activated macrophage^10^. OT is also involved in the modulation of immune and inflammatory response. Specifically, OT could decrease the release of IL-1β and IL-6 *in vitro*^20^ and *in vivo*^21,22^. Moreover, OT could also regulate the immune response by increasing levels of anti-inflammatory cytokines, such as IL-4 and IL-10^22^. These data indicated that OT might be a potential therapeutic agent for alleviating LPS-induced ALI.

The results of the present study showed that OT could attenuate LPS-induced ALI in mice. The study also found that OT inhibited the production of inflammatory cytokines in BALF. Besides, Western blot analysis, IHC, and IF all showed that OT significantly inhibited NLRP3 and NF-κB expression. Moreover, the upregulation of expression OTR on the alveolar macrophages (shown in Supplementary Information Fig. 2) may contribute to the anti-inflammatory properties of OT. But compared to control group, endogenous OT level decreased in response to LPS, it is likely that the anti-inflammatory effects mostly were from exogenous OT. These results indicated the positive effects of exogenous OT on LPS-induced ALI.

In ALI, neutrophils are the earliest immune cells recruited to the site of injury, which generate cytotoxic products^23^. A previous study showed that the elimination of neutrophils could relieve the severity of ALI^3^. MPO activity, a marker of neutrophil influx into tissues, is measured for quantifying neutrophil accumulation in tissues^24^. This study found that OT significantly inhibited MPO activity. In addition, LPS-induced lung histopathological injury was also inhibited by OT. Pulmonary edema is a characteristic of ALI. The lung W/D ratio was measured to assess the magnitude of pulmonary edema. The study showed that OT significantly decreased the lung W/D ratio, indicating a significant inhibition of edema in lung tissue. However, L-368,899 could obviously weaken these effects of OT to increase MPO activity and W/D ratio, and increased the level of OTR and OT. Meanwhile, our experiment showed that L-368,899 significantly exacerbates inflammation compared LPS group. These data suggested that intraperitoneal oxytocin’s anti-inflammatory effects were possible due to its binding to OTR, because the anti-inflammatory effects on LPS-induced ALI were effectively blocked by the OTR antagonist L-368,899.

A complex network of cytokines, including IL-1β, IL-6, and IL-18, mediates the inflammatory response in LPS-induced ALI^25^. These cytokines are released mainly from LPS-stimulated monocytes and macrophages, and recruit neutrophils into lung tissues, which is vital for host defense and contributes to the development of ALI^26^. Furthermore, IL-4 and IL-10, which are potent anti-inflammatory cytokines, could suppress the activity of many inflammatory molecules^27,28^. Two signaling pathways are required for the maturation and secretion of IL-1β and IL-18: NF-κB and NLRP3 inflammasome^29^. NF-κB, an important transcription factor, is vital in regulating the inflammatory mediators^30^. Upon exposure to LPS, activated NF-κB could regulate the expression of pro-IL-1β and pro-IL-18. NLRP3 inflammasome, an important factor in innate immunity, regulates the maturation and secretion of IL-1β and IL-18. Activated NLRP3 inflammasome leads to the activation of caspase-1, which induces the secretion and maturation of IL-1β and IL-18^31^. In addition, previous studies showed that inhibiting the NLRP3 signaling pathway could attenuate LPS-induced ALI^31,32^. The effects of OT on NF-κB expression and NLRP3 inflammasome activation were detected to investigate the mechanism underlying the anti-inflammatory effect of OT. OT dramatically decreased the expression of these cytokines and inhibited NF-κB expression and NLRP3 inflammasome activation. In contrast, OT increased the expression of anti-inflammatory cytokines, such as IL-4 and IL-10. However, L-368,899 could obviously weaken the aforementioned effects of OT. These observations further confirmed the protective and therapeutic effects of OT combined with OTR against LPS-induced ALI.

TLR4, a pattern recognition receptor, could detect LPS from Gram-negative bacteria^33^. A previous study showed that LPS–TLR4 signaling not only activated NLRP3 inflammasome and release of IL-1β, but also upregulated IL-1RI expression through NF-κB-dependent signaling^34^. The present study showed that OT inhibited TLR4 expression; however, L-368,899 increased TLR4 expression.

In conclusion, the present study demonstrated that OT combined with OTR had protective effects against LPS-induced ALI by inhibiting the inflammatory response in mouse models of LPS-induced ALI. The mechanism might involve blocking the activation of TLR4/NLRP3/NF-κB signaling pathway. These data provided evidence supporting the idea that OT represented as a novel therapeutic strategy against ALI.

## Materials and Methods

### Materials

LPS (*Escherichia coli*, O55:B5) and oxytocin receptor (OTR) antagonist L-368,899 (purity ≥ 98%) were purchased from Sigma (MO, USA). OT was purchased from ApexBio (TX, USA). A myeloperoxidase (MPO) assay kit was purchased from Cusa Biotech (Wuhan, China). All enzyme-linked immunosorbent assay (ELISA) kits of cytokines were purchased from Dake Wei (Beijing, China), and OT ELISA kits was purchased from Elabscience (Wuhan, China).

### Animals

Male C57BL/6 mice (aged 6–8 weeks, weighing 20–22 g) were purchased from the Experimental Animals Center of Shandong University (Jinan, China). The experimental protocol for all mice was approved by the Medical Ethics Committee for Experimental Animals of Shandong University (Number ECAESDUSM 2012029). We also confirmed that all methods were performed in accordance with the relevant guidelines and regulations. The animals were housed in a temperature-controlled room with a 12-h day/night cycle at 25 °C, and had free access to food and water.

After adaption for a week, all mice were randomly divided into four groups (n = 9 in each group), and all drugs were injected intraperitoneally (ip): control group (90 min saline +30 min saline + saline), LPS group [90 min saline +30 min saline + LPS (10 mg/kg, 2 mg/mL)], LPS + OT group [90 min saline +30 min OT (0.1 mg/kg, 10 µg/mL) + LPS (10 mg/kg, 2 mg/mL)], and LPS + OT + L-368,899 group [90 min L-368,899 (5 mg/kg, 1 mg/mL) +30 min OT (0.1 mg/kg, 10 µg/mL) + LPS (10 mg/kg, 2 mg/mL)]. The doses of L-368,899 used in this study were based on a previous study^35^. Two hours after LPS treatment, the mice were anesthetized by an intraperitoneal (ip) injection of 50 mg/kg sodium pentobarbital. Then, the BALF and lung tissues were collected for further analyses.

### Histopathological assessment with hematoxylin and eosin staining

The right upper lung was collected and fixed in 4% paraformaldehyde. Then, the tissues were embedded in paraffin, cut into 4-µm sections, and stained with hematoxylin and eosin (HE). Finally, the pathological changes in lung tissues were observed using an optical microscope. The histological scoring parameters included edema, intra-alveolar cell infiltration, congestion and alveolar hemorrhage. The score of each item was recorded as 1 of the following 4 grades: normal(0), mild^1^, moderate^2^; and severe^3^.

### Measurement of MPO activity in BALF

MPO activity, a biochemical marker for the infiltration of neutrophils and macrophages into the lungs, was measured using test kits purchased from Wuhan Huamei Biotechnology Institute, China. MPO activity in BALF was assayed using the MPO assay kit according to the manufacturer’s protocol. The enzymatic activity was assessed at 450 nm using a Varioskan Flash multifunction plate reader (Thermo Scientific, IL, USA).

### Measurement of lung wet/dry ratio

The severity of pulmonary edema was assessed by the wet/dry ratio (W/D ratio). The wet weight of right lower lungs was measured, and then the lungs were incubated in an oven (60 °C for 72 h) to obtain a dry weight. Finally, the W/D ratio was calculated using the wet and dry weights.

### Measurement of cytokines levels in BALF

Mouse BALF was used to measure the levels of inflammatory cytokines, such as IL-1β, IL-18, IL-6, IL-4, and IL-10. These cytokines were measured using ELISA kits following the manufacturer’s protocol. The optical density of the microplate was read at 450 nm using a Varioskan Flash multifunction plate reader (Thermo Scientific).

### Real-time quantitative polymerase chain reaction analysis

The levels of relative genes were measured by quantitative polymerase chain reaction (qPCR). The total RNA from lung tissues was extracted using kits according to the manufacturer’s protocol (Aidlab Biotech, China). The cDNA was synthesized using the SYBR Green PCR Kit (Toyobo, Japan). Subsequently, the fluorescent quantification of relative genes was performed using the Bioer Real-Time qPCR System (Bioer Technology, China) with a 10-µL reaction system comprising the following: cDNA, 1 µL; forward primers, 0.1 µL; reverse primers, 0.1 µL; mix (TaKaRa, Japan), µL; and nuclease-free water, 3.8 µL. The specific sequences of primers used in the present study for gene amplification are shown in Table 1. The primers were obtained from Beijing Genomics Institute (Beijing, China). The cycling conditions were as follows: 95 °C for 30 s, 95 °C for 10 s, 56 °C for 10 s, and 72 °C for 30 s. The expression levels of targeting mRNA levels were normalized to actin.

**Table 1:** Primer sequences of RT-qPCR test.

Gene	Forward primers(5′-3′)	Reverse primers(5′-3′)
IL-1β	ATGAAAGACGGCACACCCAC	AAGGCAGAGTCTTCGGTGAG
IL-6	CAACCAAGAGGTGAGTGCTTC	GGTGTCCTCTTTCCCACACTG
IL-18	GGAAGACCAGAGACATCCACTG	ACACTAGACCAAAGGGCTTG
IL-4	CTGTAGGGCTTCCAAGGTGC	CTCTCATTGTGCCAGGTCACT
IL-10	GCCAGTTAGAAAGCCACCAC	GGTTCAGCCTGTTTCCCAAC
ACTIN	GGCTGTATTCCCCTCCATCG	CCAGTTGGTAACAATGCCATGT

IL, Interleukin.

### Western blot analysis

The total protein of lung tissue was extracted by adding cold radioimmunoprecipitation assay buffer (Beyotime Institute of Biotechnology, Shanghai, China) and using an electric homogenizer to obtain supernatant fluid after centrifuging the homogenates at 12,000 rpm for 20 min at 4 °C. As previously described, protein concentrations were assessed using a bicinchoninic acid protein assay kit (ComWin Biotech, China). Subsequently, supernatants in equal volumes of sample buffer were boiled for 10 min at 95 °C and loaded in 10% and 12% gels for sodium dodecyl sulfate–polyacrylamide gel electrophoresis, followed by transfer to a polyvinylidene difluoride membrane (Millipore, MA, USA). After blocking the nonspecific site with a blocking solution (5% nonfat dry milk) for 1 h at room temperature, the membranes were washed in Tris-buffered saline with Tween 20 (TBST) three times and incubated overnight at 4 °C with specific primary antibodies. The primary antibodies were rabbit anti-OTR (dilution 1:1000, Abcam, Cambridge, UK), rabbit anti-TLR4 (dilution 1:1000, Abcam), rabbit anti-NLRP3 (dilution 1:1000, Abcam), rabbit anti-caspase-1 (dilution 1:1000, Abcam), rabbit anti-nuclear factor-κB (dilution 1:1000, Cell Signaling Technology, MA, USA), rabbit anti-IL-1β (dilution 1:1000, Abcam), rabbit anti-IL-6 (dilution 1:1000, Abcam), and rabbit anti-actin (dilution 1:2000, Proteintech Group, Wuhan, China). Then, all membranes were washed in TBST three times before incubating with horseradish peroxidase–conjugated secondary antibody (dilution 1:2000, ZSGB-BIO, China) for 1 h at room temperature. Finally, the membranes were visualized with enhanced chemiluminescence (ComWin Biotech, China) and imaged using a BioRad Chemi-Doc gel system after an additional wash.

### Immunohistochemistry

Paraffin-embedded lung sections used for immunostaining were dewaxed and exposed to citrate buffer for antigen unmasking. The sections were then blocked for 30 min at room temperature and incubated overnight at 4 °C with specific primary antibodies. The primary antibodies were rabbit anti-OTR (dilution 1:500, Abcam), rabbit anti-TLR4 (dilution 1:500, Abcam), rabbit anti-NLRP3 (dilution 1:400, Abcam), rabbit anti-caspase-1 (dilution 1:400, Abcam), rabbit anti-NF-κB (dilution 1:500, CST), rabbit anti-IL-1β (dilution 1:500, Abcam), and rabbit anti-IL-6 (dilution 1:800, Abcam). A VECTASTAIN Universal Quick Kit, Ready-to-Use Kit (Vector Laboratories, CA, USA) was used as a source of secondary antibodies. The mounted tissues were stained with 3,3-diaminobenzidine (DAB) and observed under a Nikon Eclipse 80i light microscope. All the images were quantified using ImageJ.

### Immunofluorescence (IF)

After dewaxing and antigen unmasking, the sections were then blocked for 1 h with 10% goat serum albumin solution at room temperature and incubated overnight at 4 °C with specific primary antibodies. The primary antibodies were rabbit anti-OTR (dilution 1:500, Abcam), rabbit anti-TLR4 (dilution 1:200, Abcam), rabbit anti-NLRP3 (dilution 1:200, Abcam), rabbit anti-caspase-1 (dilution 1:400, Abcam), rabbit anti-NF-κB (dilution 1:500, CST), rabbit anti-IL-1β (dilution 1:300, Abcam), and rabbit anti-IL-6 (dilution 1:500, Abcam). After incubation, the sections were washed three times with phosphate-buffered saline (PBS) successively for 5 min each. The sections were then incubated with the fluorescein isothiocyanate–conjugated secondary antibody (1:2000 dilution in PBS with 1.5–3% bovine serum albumin) in a moist dark chamber for 2.5 h at room temperature. The sections were washed again in the same manner as described earlier and counterstained with 2-(4-amidinophenyl)-6-indolecarbamidine dihydrochloride for 5 min at room temperature, followed by a washing step with PBS as earlier. The sections were then covered with glass coverslips and observed under a Nikon Eclipse 80i light microscope. All the images were quantified using ImageJ.

### Measurement of OT levels in lung tissue

The total protein of lung tissue was extracted and concentrations were assessed in the same manner as described as Western blot. OT was measured using ELISA kits following the manufacturer’s protocol. The optical density of the microplate was read at 450 nm using a Varioskan Flash multifunction plate reader (Thermo Scientific).

### Statistical analysis

Student’s t-test was performed for paired samples. For data that were not normally distributed (such as cytokines), multiple comparisons were carried out using Kruskal-Wallis test. All results were presented as the means ± standard error of the mean and analyzed using Sigmaplot software (version 20.0 for Windows; SPSS Inc. IL, USA). A *P* value < 0.05 was considered to indicate a statistically significant difference.

## Supplementary information

Supplementary Information

## Data Availability

The data set supporting the results of this article are included within the article.
