# Supplementary material for: Protective effect of oxytocin on LPS-induced acute lung injury in mice
Source: Sci Rep. 2019 Feb 26;9:2836. doi: 10.1038/s41598-019-39349-1 (PMC6391417; doi:10.1038/s41598-019-39349-1)

Protective effect of oxytocin on LPS-induced acute lung injury in mice
Xiaona An, Xiaotong Sun, Yonghao Hou, Xiaomei Yang, Hongli Chen, Peng Zhang, Jianbo Wu

The expression levels of IL-6, OTR, NF-κB, and NLRP3 were detected using Western blot analysis to further explain how OT–OTR affected inflammation. As shown in Figure 1, L-368,899 significantly exacerbated inflammation compared with the LPS group (LPS group had significant inflammation compared with the control). These data suggested that the anti-inflammatory effects of intraperitoneal oxytocin were possible due to its binding to OTR. The anti-inflammatory effects on LPS-induced ALI were effectively blocked by the OTR antagonist L-368,899.

Previous studies demonstrated that the expression of OTR increased in LPS-activated macrophages (1). The present study also showed an increase in the protein level of OTR compared with the control group. Figure 2 shows that OTRs were mainly expressed on the alveolar macrophages, as shown in Figure 2.


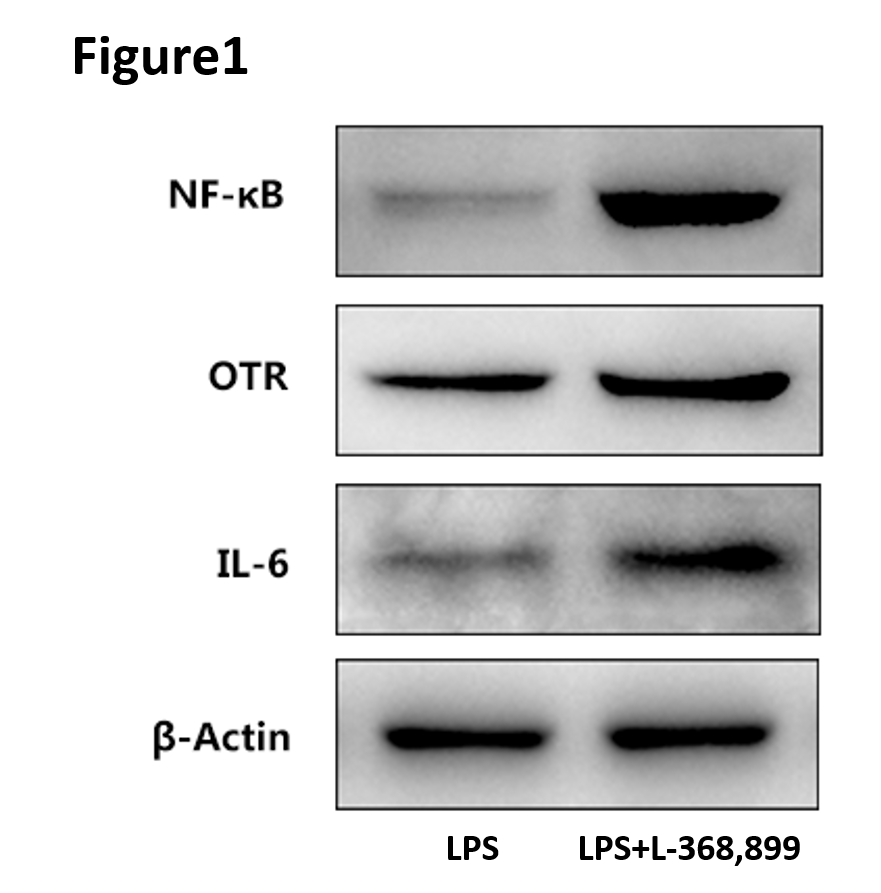


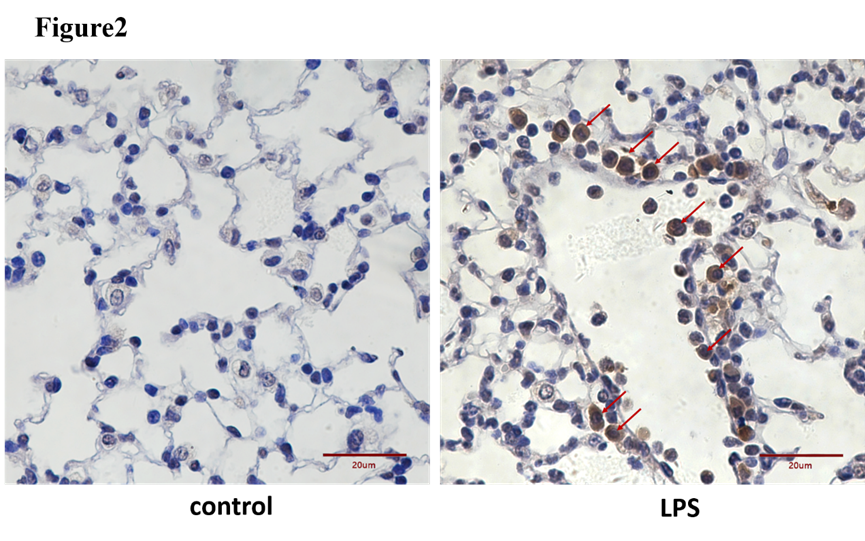

Supplement: Supplementary file 1 — Supplementary Information [file 41598_2019_39349_MOESM1_ESM.docx]
